# Perceived vs. Actual Emotion Reactivity and Regulation in Individuals With and Without a History of NSSI

**DOI:** 10.3389/fpsyg.2021.612792

**Published:** 2021-03-01

**Authors:** Jessica Mettler, Melissa Stern, Stephen P. Lewis, Nancy L. Heath

**Affiliations:** ^1^Department of Educational and Counselling Psychology, McGill University, Montreal, QC, Canada; ^2^West Island Neuropsychology and Counseling Centre, Montreal, QC, Canada; ^3^Department of Psychology, University of Guelph, Guelph, ON, Canada

**Keywords:** differences in experimental vs. *in vivo*, emotion reactivity, emotion regulation, positive emotions, negative emotions

## Abstract

Non-suicidal self-injury (NSSI) has consistently been associated with self-reported difficulties in emotion reactivity and the regulation of negative emotions; however, less is known about the accuracy of these self-reports or the reactivity and regulation of positive emotions. The present study sought to investigate differences between women with and without a history of NSSI on: (a) self-reported general tendencies of negative and positive emotion reactivity, (b) self-reported general tendencies of negative and positive emotion regulation, and (c) emotion regulation reported in response to a positive and negative mood induction. The sample consisted of 36 women with a recent history of NSSI within the last 2 years (*Mage* = 20.06; *SD* = 1.51) and a comparison group with no history of NSSI (*n* = 34; *Mage* = 20.15; *SD* = 1.54). Participants completed self-report measures of negative and positive emotion reactivity and regulation. In a separate session, participants underwent both a negative and positive mood induction using a counterbalanced design and reported their experienced emotions. Results from two-way MANOVAs and ANOVAs revealed those with a history of NSSI reported significantly greater difficulties in negative emotion reactivity and regulation than the no-NSSI comparison group; however, no group differences emerged in self-reported reactivity or regulation of positive emotions. In contrast, repeated measures ANOVAs on data from the mood induction task found no group differences in reactivity or regulation for either negative or positive emotions. These findings highlight the possibility that although individuals with a history of NSSI evaluate their ability to manage negative emotions as significantly worse than individuals with no history of self-injury, this may not reflect their actual emotion regulatory processes.

## Introduction

Non-suicidal self-injury (NSSI) is the deliberate damaging of body tissue without the intent to die and for purposes not socially sanctioned (International Society for the Study of Self-Injury, 2007; Nixon and Heath, 2009). According to the International Society for the Study of Self-Injury (ISSS) and researchers in the field, NSSI includes behaviors such as cutting, scratching, self-hitting, and burning, but excludes extreme tattooing or body piercing (International Society for the Study of Self-Injury, 2007; Nock and Favazza, 2009). Although NSSI prevalence rates seem to vary according to age groups, they are consistently high amongst university students, with rates ranging from 15 to 39%, thus making the study of NSSI behaviors in this age group particularly important (e.g., Swannell et al., 2014; Cipriano et al., 2017). Interestingly, NSSI is also a prevalent behavior within both community and clinical populations, typically emerging in adolescence and associated with an increased risk of suicide and mental health difficulties such as depression and anxiety (e.g., Klonsky et al., 2003; Muehlenkamp and Gutierrez, 2007; Swannell et al., 2014).

The present study was conducted to investigate differences in self-reported and actual emotion regulatory processes for both negative and positive emotions between those with and without a history of NSSI engagement. Indeed, one of the most commonly endorsed motivations for engaging in NSSI is to regulate negative emotions [e.g., see review by Taylor et al. (2019)]; therefore, most of the existing literature has focused on investigating the role of negative emotions in the development and maintenance of NSSI and there is much less research investigating the role of positive emotions in NSSI engagement (e.g., Adrian et al., 2011; Jenkins and Schmitz, 2012). However, investigating the influence of positive emotions provides a new lens for NSSI research given that positive and negative emotions have been shown to be differentially associated with mental health, well-being, and health outcomes (e.g., Moskowitz et al., 2019; Pressman et al., 2019). Specifically, within the field of NSSI research, recent evidence suggests that positive emotions are differentially associated with NSSI engagement depending on the degree to which negative affect is also reported (e.g., Hasking et al., 2018). These findings are in line with Frederickson's broaden-and-build theory of positive emotions, which suggests that the presence of positive emotions can in and of itself be protective and functions differently from negative emotions (e.g., Fredrickson, 2013). Thus, further research is needed to investigate the role of positive emotions in NSSI engagement. Finally, beyond being focused on negative emotions, almost all research on emotion reactivity and regulation has relied on self-report methods that may be influenced by a recall bias rather than assessing actual regulatory processes. Therefore, there is a need to extend NSSI research to investigate positive emotions and to compare self-report with *in vivo* emotion regulatory processes.

One of the factors that may influence an individual's ability to regulate emotions is emotion reactivity, which is defined as individual differences in the intensity and temporal nature of behavioral or physiological responses to emotional stimuli (Chapman et al., 2006; Rothbart et al., 2011). It comprises 3 components: (a) the extent to which an individual experiences emotions in response to stimuli (i.e., emotion sensitivity), (b) how strongly or intensely the emotional experience is (i.e., emotion intensity), and (c) the period of time needed to return to a baseline level of arousal (i.e., emotion persistence; Nock et al., 2008). Furthermore, emotion reactivity is believed to be stable across emotional valence: thus, according to theory, individuals who experience strong positive emotions will also experience strong negative emotions (Larsen and Diener, 1987).

Past research suggests that individuals who engage in NSSI report higher levels of emotion reactivity for negative emotions (e.g., Nock et al., 2008; Jenkins and Schmitz, 2012). For example, a study conducted by Baetens and colleagues (Baetens et al., 2011) revealed that adolescents who engage in NSSI are more likely to report greater levels of negative affect and frustration compared to individuals who do not engage in NSSI. Similarly, Anderson and Crowther (2012) found that undergraduate students who had a history of NSSI had more intense negative emotional experiences compared to those who had never engaged in NSSI.

Beyond emotion reactivity, it is also important to consider how individuals regulate their emotions in order to manage their emotion reactivity (e.g., Gross and John, 2003). Research is consistent in suggesting individuals with a history of engaging in NSSI have difficulties regulating their negative emotions (e.g., Richmond et al., 2015; Nicolai et al., 2016; Zelkowitz et al., 2017). Furthermore, research shows the NSSI engagement is itself often used as a means of down-regulating negative emotions and up-regulating positive emotions (e.g., Klonsky, 2009; Claes et al., 2010).

Although most research has focused on the association between NSSI and challenges in emotion reactivity and regulation of negative emotions (e.g., Adrian et al., 2011; Turner et al., 2012), much less has focused on examining the association between positive emotions and NSSI engagement. Further, the few studies that have investigated the role of positive emotions in NSSI engagement focused on the general experience of positive emotions, such as the frequency with which positive emotions occur (e.g., Victor and Klonsky, 2014), and the removal of negative emotions (i.e., calm and relief) following engagement in NSSI (e.g., Klonsky, 2009; Claes et al., 2010). Interestingly, recent evidence suggests that the emotion regulatory function of NSSI for negative emotions may depend on the frequency with which individuals experience positive emotions. Specifically, experiencing positive emotions may be protective against NSSI when experiencing intense negative emotions but may lead to increased NSSI engagement when experiencing low negative affect (Hasking et al., 2018). Accordingly, further research is warranted to investigate the unique influence of positive and negative emotions on NSSI engagement in terms of the experience of emotions as well as their reactivity and regulation.

Moreover, much of the research on NSSI engagement and emotion reactivity and regulation has been conducted either through retrospective reports or diary studies where individuals answer questions pertaining to their emotional experiences as soon as they are able to following the event (e.g., Adrian et al., 2011; Victor and Klonsky, 2014). Unfortunately, these studies very rarely target emotions as the individual is actively experiencing them. Nevertheless, efforts have been made to examine individuals' current mood state in order to identify potential differences between the emotional experiences of those with and without a history of NSSI. Here, mood induction techniques have shown utility as they account for emotions in real time (e.g., Bresin and Gordon, 2013; Arbuthnott et al., 2014).

Overall, very few studies have used mood induction to examine the reactivity and regulation of emotions compared to individuals without a history of NSSI but findings so far have been mixed. Specifically, Davis and colleagues (Davis et al., 2014) conducted a negative mood induction using a sad movie clip and did not find significant differences in reports of negative emotion reactivity between individuals with a history of NSSI, a clinical control group without a history of NSSI matched on symptoms of anxiety and depression, and a control group with no history of anxiety, depression, or NSSI. In contrast, another study found different results indicating that individuals with a history of NSSI self-reported significantly greater emotion reactivity than those without such a history (Glenn et al., 2011). However, unlike Davis and colleagues (Davis et al., 2014), Glenn and colleagues (Glenn et al., 2011) used a mood induction combining negative, neutral, and positive images rather than a negatively valenced video clip. Therefore, further research is needed to investigate the unique outcomes from either negative or positive mood inductions.

Furthermore, there is a paucity of mood induction studies assessing both negative and positive emotions as outcomes in NSSI research. Using a rumination induction, Arbuthnott and colleagues (Arbuthnott et al., 2014) assessed both negative and positive affect and found that individuals with a history of NSSI reported significantly greater increases in negative affect during the task when compared to a comparison group of individuals with eating disorders, whereas the comparison group reported greater decreases in positive emotions than those with a history of NSSI. These results suggest that positive and negative emotions may be differentially affected by a negative emotional situation for individuals with distinct difficulties (such as those who engage in NSSI compared to those with an eating disorder).

In a related study, Boyes and colleagues (Boyes et al., 2020) used both a negative and a positive mood induction and found that individuals with a history of NSSI displayed significantly less emotion reactivity for both negative and positive emotions than those without such a history following the mood inductions. However, the authors did not investigate group differences for both negative and positive emotions within each mood induction valence type. Rather, only negative emotions were assessed during the negative mood induction and only positive emotions were assessed during the positive mood induction. Given that positive and negative emotions have been shown to be non-mutually exclusive and to have differential outcomes and mechanisms, there is a need for a more in-depth investigation of how positive and negative emotions are affected within each valence of mood induction.

Therefore, the present study seeks to address the lack of research on self-reported positive emotion reactivity and regulation for individuals with a history of NSSI engagement and the promising findings in the area of mood induction research for NSSI. The objectives of the current study were to investigate differences between individuals with and without a history of NSSI engagement in terms of (a) self-reported emotion reactivity, (b) self-reported emotion regulation, and (c) actual emotion regulatory processes in response to negative and positive mood inductions. Each of these objectives will be examined first with a focus on negative emotions and then with a focus on positive emotions.

The first objective was to investigate differences in self-reported emotion reactivity. It was hypothesized that individuals engaging in NSSI would self-report significantly greater difficulty with emotion reactivity for both negative (H1a, i.e., report greater reactivity to negative emotions) and positive (H1b, i.e., report less reactivity to positive emotions) emotions than the non-NSSI group.

The second objective was to investigate differences in self-reported emotion regulation. It was hypothesized that the NSSI group would self-report significantly less success in emotion regulation in response to negative (H2a; i.e., less ability to down regulate negative emotions) and positive (H2b; i.e., less ability to up regulate positive emotions) emotions compared to the non-NSSI group.

Finally, the third objective was to investigate actual emotion regulatory processes in response to both a negative and a positive mood induction. Based on previous findings by Arbuthnott and colleagues (Arbuthnott et al., 2014), it was hypothesized (H3) that individuals with a history of NSSI would report higher levels of negative affect in response to both the negative and positive mood induction than individuals who have never engaged in NSSI. In terms of positive affect, it was hypothesized (H4) that individuals with a history of NSSI would report significantly less positive affect than those without a history of NSSI in response to both the negative and positive mood inductions. Interactions were also expected (H5) such that individuals with a history of NSSI would require significantly more time to recover (i.e., return to baseline) from negative emotions in response to the negative mood induction and less time to return to baseline from positive emotions in response to the positive mood induction than individuals in the non-NSSI group.

## Materials and Methods

### Participants

Participants were female undergraduate students (*N* = 74) recruited from a large urban Canadian university using two recruitment methods. First, following IRB approval, a research team database was used to contact individuals who had agreed to be contacted about participation in studies on stress and coping and who had previously completed a screening questionnaire pertaining to their NSSI engagement. Participants were also recruited from an advertisement posted on the university's online classifieds and social media pages.

As a result of data cleaning, 4 participants had to be removed from the study (details are provided in the Result section below); thus, the final sample consisted of 36 female participants who reported a history of NSSI engagement over the past 2 years (*M*age = 20.06 years; *SD* = 1.51), as well as a comparison group consisting of 34 female participants with no history of NSSI engagement (*Mage* = 20.15 years; *SD* = 1.54). Table 1 presents the sample's demographic information.

**Table 1:** Sample demographics.

**Ethnicity (*****N*** **=** **70)**		**NSSI Frequency** ***(n*** **=** **36)**	
Caucasian	57.1%	Once	5.6%
Asian	28.6%	2–4 times	11.1%
Other	8.6%	5–10 times	8.3%
Mixed	5.7%	11–50 times	50%
		51–100 times	8.3%
		100 times or more	16.7%

### Measures

#### NSSI Screening Questionnaire

A self-report researcher-designed questionnaire assessing stress and coping in university students was administered campus-wide as part of a previous study. NSSI is included as one of the listed coping behaviors that participants have the option to choose from (i.e., “physically hurt myself on purpose without wanting to die”). This screener questionnaire also included a question asking whether participants were interested in being contacted again about future studies with our team. Therefore, the NSSI engagement item was used to provide preliminary information to identify a subsample of individuals who may either be currently engaging in NSSI or have a history of NSSI engagement and these people were sent the invitation email to participate in the present study.

#### Non-suicidal Self-Injury

The Inventory of Statements about Self-Injury (ISAS; Klonsky and Glenn, 2009) is a self-report measure that assesses various aspects of NSSI, with sections assessing the frequency and functions of NSSI. For the purpose of this study, only information relating to the frequency of NSSI was used. This measure was only administered to individuals who indicated that they had ever engaged in NSSI on the NSSI screening questionnaire described above, in order to confirm NSSI engagement and specifically identify individuals who had engaged in NSSI over the past 2 years.

#### Emotion Reactivity

All participants completed the Emotion Reactivity Scale (ERS; Nock et al., 2008), a 21-item questionnaire developed to assess how individuals experience emotions. In particular, the ERS assesses 3 aspects of emotion reactivity including: (a) sensitivity (e.g., “even the littlest things make me emotional”), (b) intensity (e.g., “when I experience emotions, I feel them very strongly/intensely”), and (c) persistence (e.g., “when something happens that upsets me, it's all I can think about for a long time”). For the purpose of this study, the ERS was also adapted to assess positive emotions by adding questions that are the positive emotion equivalents for each item (e.g., “when something happens that makes me happy, it's all I can think about for a long time”). In the present study, the internal consistency of the ERS was good both for negative (Cronbach's α: sensitivity = 0.92; intensity = 0.92; persistence = 0.80) and positive (Cronbach's α: sensitivity = 0.86; intensity = 0.84; persistence = 0.77) emotion reactivity.

#### Emotion Dysregulation

The Regulatory Emotional Self-Efficacy scale (RESE; Caprara and Gerbino, 2001) is a well-validated 12-item self-report measure designed to assess one's efficacy in regulating negative (despondency and anger) and positive (including happiness, joy, and contentment) affect (Alessandri et al., 2015). In the current study, the Cronbach's alphas were 0.68 for despondency, 0.65 for anger, and 0.67 for positive emotions, which are deemed acceptable for research (Meyers et al., 2013).

#### Positive and Negative Emotional Experiences

The Positive and Negative Affect Scale (PANAS; Watson et al., 1988) is a self-report measure designed to assess the frequency with which an individual has experienced negative and positive emotions in the past day or week. Responses for each emotion are rated on a five-point Likert scale ranging from “very slightly or not at all” to “extremely.” The PANAS demonstrates good internal consistency, test-retest reliability, as well as convergent and divergent validity (Watson et al., 1988; Jenkins and Schmitz, 2012). Unfortunately, the internal consistency of the PANAS within this study could not be calculated due to corruption of the raw data for this instrument. For the purposes of this study, the intensity of state-level emotions was measured by looking at the change in responses from baseline to post-task intensity, which will be interpreted as reactivity. Recovery time was measured by assessing emotions at 1- and 2-min post video clip.

### Procedure

The study was conducted in 2 parts. First, participants completed an online survey including the measures described above, following which they received $10 as well a list of resources should they require additional support. Participants with a history of NSSI were subsequently emailed and asked if they would be interested in participating in an in-person follow up study on emotions. Individuals matched on age but with no history of NSSI were also invited to participate as a comparison group.

Immediately prior to completing the mood induction task, participants were asked to complete the PANAS (Watson et al., 1988) to assess their baseline emotions and their relative intensities. Participants underwent a positive and negative mood induction, using a randomized counterbalanced design whereby they were either presented with a negative video (in which a cat was trying to revive another cat lying motionless on the ground with sad background music) or a positive video (in which a young boy humorously reports on “10 things that we should say more often”). These videos were chosen in the present study based on findings from Zhang and colleagues (Zhang et al., 2014) where videos containing both affectively-congruent images and music were the most effective out of 4 types of mood inductions at inducing either a positive or negative mood. Both negative and positive video clips (each about 3 min long) had been piloted with research team volunteers prior to starting data collection to ensure the appropriate mood was induced and to determine the typical timeframe for a return to baseline for both positive and negative emotions.

Immediately following the first mood induction, participants were asked to complete the PANAS again, wait 2 min, and complete the PANAS once more. Participants then underwent a distractor task consisting of simple math problems to be solved without a time limit before completing another baseline assessment of their emotions using the PANAS prior to the second mood induction. Then, participants viewed their second mood induction video followed by a repeat of the PANAS at a 2-min interval, a distraction task, and a final completion of the PANAS to ensure a return to baseline. If a participant's mood was worse than it was at baseline (i.e., they felt more negative affect or less positive affect), they watched a humorous clip from the television show “Friends” before completing the PANAS again. The session concluded when the participant's mood was comparable to their baseline negative and positive affect.

### Analytic Plan

The first objective was to investigate differences in self-reported emotion reactivity for both negative (H1a) and positive (H1b) emotions between individuals with and without a history of NSSI. Given that the ERS (Nock et al., 2008) has 3 subscales, separate one-way MANOVAs were used with negative and positive emotion reactivity as outcomes, respectively.

The second objective was to investigate differences in self-reported emotion regulation for both negative (H2a) and positive (H2b) emotions between individuals with and without NSSI engagement. Given that the RESE (Caprara and Gerbino, 2001) has 2 subscales assessing negative emotion regulation and 1 subscale assessing positive emotion regulation, differences in regulation of negative emotions were assessed with a one-way MANOVA while differences in regulation of positive emotions were assessed with a one-way ANOVA.

Finally, the third objective was to compare differences in actual emotion regulatory processes in response to mood inductions in individuals with and without a history of NSSI. Given that each participant underwent two mood inductions (one negative and one positive) and that both negative and positive emotions were assessed before and after each mood induction, 4 separate 2 (Group: NSSI vs non-NSSI) X 4 (Time: pre, post, 1 min post, 2 min post) repeated measures ANOVAs were conducted.

## Results

All analyses were conducted using SPSS version 24. Prior to the main analyses, we evaluated patterns of missingness and cleaned the data. One participant was removed from the sample given that NSSI status had not been reported. As per recommendations by Tabachnick and Fidell (2012), the data were assumed to be missing completely at random (MCAR) given that <5% of data points were missing per variable. Therefore, the expectation maximization procedure was used to impute missing values within each measure or subscale of both the NSSI and non-NSSI groups separately to increase the accuracy of the prediction. Following imputation, 1 participant in the non-NSSI group was identified as an outlier on emotion reactivity (i.e., more than 3 SDs from the mean) and was thus excluded from the final sample. Given that all other participants were women, a participant in the non-NSSI group who reported being male was also excluded from final analyses along with a randomly selected age-matched participant in the NSSI group. Therefore, the final sample consisted of 36 female participants with a history of NSSI over the past 2 years (*M*age = 20.06 years; *SD* = 1.51) and 34 female participants without a history of NSSI (*Mage* = 20.15 years; *SD* = 1.54).

The first objective was to compare women with and without a history of NSSI in terms of their self-reported reactivity to positive and negative emotions. Separate one-way MANOVAs were conducted to test whether women with a history of NSSI would report greater difficulties with emotion reactivity for negative emotions (H1a) and positive emotions (H1b) than those without a history of NSSI. Table 2 presents the means and standard deviations for emotion reactivity of positive and negative emotions. Consistent with H1a, women with a history of NSSI reported significantly greater difficulties with emotion reactivity for negative emotions compared to those without a history of NSSI, Wilk's Λ = 0.81, *F*_(3,66)_ = 5.15, *p* = 0.003, $\eta_{\text{p}}^{2}$ = 0.19. Specifically, they reported higher levels of sensitivity, *F*_(1,68)_ = 14.87, *p* < 0.001, $\eta_{\text{p}}^{2}$ = 0.18, intensity, *F*_(1,68)_ = 10.03, *p* = 0.002, $\eta_{\text{p}}^{2}$ = 0.13, and persistence, *F*_(1,68)_ = 12.07, *p* = 0.001, $\eta_{\text{p}}^{2}$ = 0.15, for negative emotions. However, contrary to H1b, no significant differences were found when a separate MANOVA was conducted for positive emotion reactivity, Wilk's Λ = 0.99, *F*_(3,66)_ = 0.15, *p* = 932, $\eta_{\text{p}}^{2}$ = 007. Further, partial eta-squared suggested a moderate to large effect size for negative emotion reactivity and a small to moderate effect size for positive emotion reactivity.

**Table 2:** Means and standard deviations for negative and positive emotion reactivity and regulation.

	**NSSI**		**Non-NSSI**	
	***M***	***SD***	***M***	***SD***
**Negative emotion reactivity**				
Sensitivity	24.26	9.96	15.53	8.92
Intensity	16.38	8.23	10.65	6.80
Persistence	9.56	4.14	6.35	3.53
**Positive emotion reactivity**				
Sensitivity	15.72	8.18	14.82	7.59
Intensity	9.97	5.83	9.65	5.41
Persistence	6.08	4.11	5.62	2.83
**Emotion regulation**				
Despondency	4.78	2.83	6.47	2.97
Anger	5.28	3.08	7.12	2.69
Positive emotions	10.47	3.08	10.85	3.05

The second objective of the present study was to investigate group differences in terms of self-reported emotion regulation for negative and positive emotions between women with and without a history of NSSI. Similarly to the first objective, a one-way MANOVA was conducted to test H2a that women with a history of NSSI would report worse emotion regulation for negative emotions. Table 2 also presents the means and standard deviations for emotion regulation of positive and negative emotions. Results revealed significant group differences at the multivariate level, Wilk's Λ = 0.88, *F*_(2,67)_ = 4.46, *p* = 0.015, $\eta_{\text{p}}^{2}$ = 0.746. Specifically, women with a history of NSSI reported worse negative emotion regulation for both despondency, *F*_(1,68)_ = 5.97, *p* = 0.017, $\eta_{\text{p}}^{2}$ = 0.08, and anger, *F*_(1,68)_ = 7.05, *p* = 0.01, $\eta_{\text{p}}^{2}$ = 0.09, compared to women without a history of NSSI, with a moderate effect size. A one-way ANOVA was then conducted to test H2b that women with a history of NSSI engagement would report worse emotion regulation for positive emotions than those without. However, contrary to H2b, no significant differences were found between those with and without a history of NSSI for emotion regulation of positive emotions, *F*_(1,68)_ = 0.27, *p* = 0.605, $\eta_{\text{p}}^{2}$ = 0.004.

**Figure 1:**
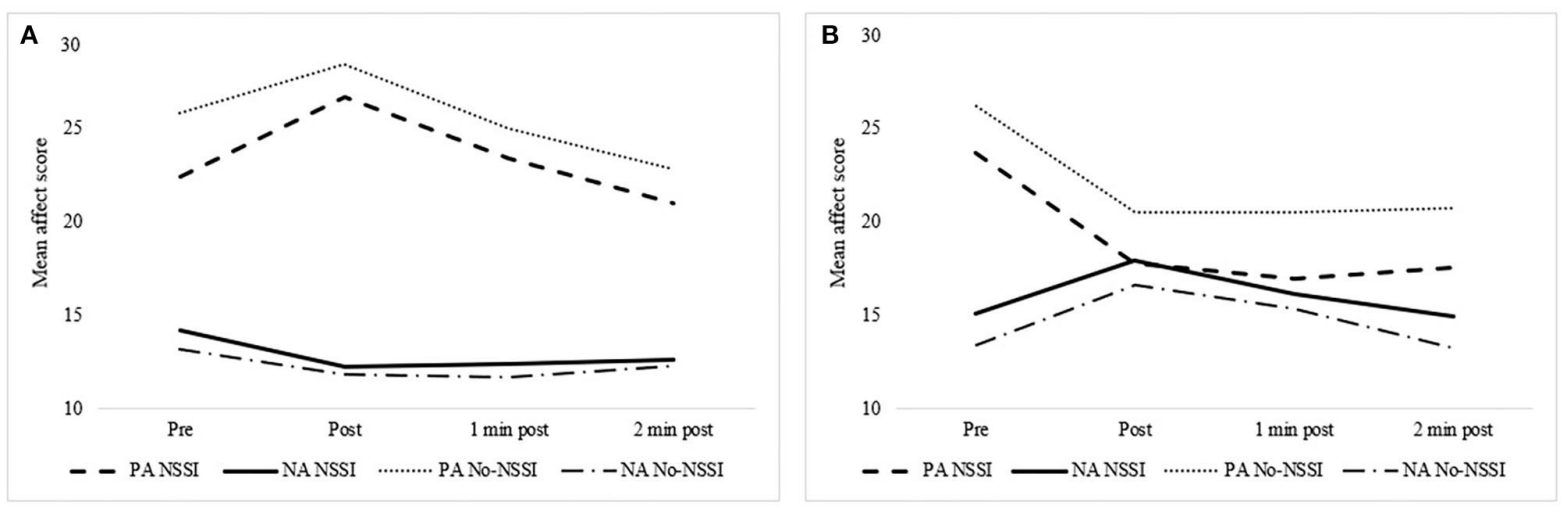
**(A)** presents the results for the positive mood inducement pre- and post-mean negative (NA; blue lines and positive (NA; orange lines) affect scores for women with and without a history of NSSI. **(B)** presents the results for the negative mood inducement pre- and post-mean negative (NA; blue lines) and positive (PA; orange lines) affect scores for women with and without a history of NSSI.

The third objective aimed to compare negative and positive emotions for women with and without a history of NSSI in response to a negative and positive mood induction. Four separate 2 (Group: NSSI vs non-NSSI) × 4 (Time: pre, post, 1 min post, 2 min post) repeated measures ANOVAs were conducted: one for each type of negative and positive emotion within each condition (negative vs. positive mood induction). Table 3 presents the means and standard deviations of negative and positive affect across all time points for the NSSI and non-NSSI groups within both the negative and positive mood inductions. Additionally, results for the repeated measures ANOVAs are presented in Table 4 and Figure 1. No significant interactions were found in any of the 4 repeated measures ANOVA analyses across the negative or positive affect and mood induction tasks.

**Table 3:** Means and standard deviations for negative and positive emotions across time points (pre, post, 1 min post, 2 min post) for NSSI and non-NSSI groups within the negative and positive mood inductions.

		**NSSI**		**Non-NSSI**	
		***M***	***SD***	***M***	***SD***
**Negative mood**	**Negative affect**				
**induction**	Pre	15.08	6.71	13.62	2.94
	Post	17.89	7.02	16.91	4.27
	1 min post	16.08	6.35	15.68	4.41
	2 min post	14.89	6.20	13.35	3.05
	**Positive affect**				
	Pre	23.50	7.28	25.65	6.94
	Post	17.94	5.94	19.94	5.44
	1 min post	16.94	5.57	19.91	6.47
	2 min post	17.50	6.22	20.21	6.67
**Positive mood**	**Negative affect**				
**induction**	Pre	14.22	4.46	13.21	3.04
	Post	12.22	2.71	11.97	2.50
	1 min post	12.36	3.14	11.82	2.52
	2 min post	12.58	3.38	12.35	2.91
	**Positive affect**				
	Pre	22.47	7.40	25.24	6.18
	Post	26.67	9.90	27.91	7.90
	1 min post	23.19	9.03	24.38	7.24
	2 min post	20.67	8.65	22.15	6.43

**Table 4:** Results of 2 (Group: NSSI vs. non-NSSI) X 4 (Time: pre, post, 1 min post, 2 min post) repeated measures ANOVAs for negative and positive affect following a negative and positive mood induction.

**Negative affect—negative mood induction**	
Mauchly's test of sphericity—Time	χ^2^ (5) = 46.71, *p* < 0.001
Interaction—*Greenhouse-Geisser*	*F*_(3,204)_ = 0.675, *p* = 0.527, $\eta_{\text{p}}^{2}$ = 0.010, 1–β = 0.17
Main effect of Time (within)—*Greenhouse-Geisser*	*F*_(2.24, 204)_ = 22.94, *p* < 0.001, $\eta_{\text{p}}^{2}$ = 0.252, 1–β = 1
Main effect of Group (between)	*F*_(1,68)_ = 0.886, *p* = 0.350, $\eta_{\text{p}}^{2}$ = 0.013, 1–β = 0.15
**Positive affect—negative mood induction**	
Mauchly's test of sphericity–Time	χ^2^ (5) = 37.53, *p* < 0.001
Interaction—*Greenhouse-Geisser*	*F*_(3,204)_ = 0.419, *p* = 0.676, $\eta_{\text{p}}^{2}$ = 0.006, 1–β = 0.12
Main effect of Time (within)—*Greenhouse-Geisser*	*F*(2.18, 204) = 68.26, *p* < 0.001, $\eta_{\text{p}}^{2}$ = 0.501, 1–β = 1
Main effect of Group (between)	*F*_(1,68)_ = 3.13, *p* = 0.081, $\eta_{\text{p}}^{2}$ = 0.044, 1–β = 0.42
**Negative affect—positive mood induction**	
Mauchly's test of sphericity–Time	χ^2^ (5) = 63.02, *p* < 0.001
Interaction—*Greenhouse-Geisser*	*F*_(3,204)_ = 0.838, *p* = 0.436, $\eta_{\text{p}}^{2}$ = 0.012, 1–β = 0.19
Main effect of time (within)—*Greenhouse-Geisser*	*F*_(2.03, 204)_ = 14.75, *p* < 0.001, $\eta_{\text{p}}^{2}$ = 0.178, 1–β = 1
Main effect of group (between)	*F*_(1,68)_ = 0.579, *p* = 0.449, $\eta_{\text{p}}^{2}$ = 0.008, 1–β = 0.12
**Positive affect—positive mood induction**	
Mauchly's test of sphericity–Time	χ^2^ (5) = 35.23, *p* < 0.001
Interaction—*Greenhouse-Geisser*	*F*_(2.4, 204)_ = 0.89, *p* = 0.431, $\eta_{\text{p}}^{2}$ = 0.013, 1–β = 0.22
Main effect of time (within)—*Greenhouse-Geisser*	*F*_(2.4, 204)_ = 37.88, *p* < 0.001, $\eta_{\text{p}}^{2}$ = 0.358, 1–β = 1
Main effect of group (between)	*F*_(1,68)_ = 0.88, *p* = 0.351, $\eta_{\text{p}}^{2}$ = 0.013, 1–β = 0.15

However, significant main effects of Time were found for each repeated measures ANOVA across negative and positive affect for both types of mood induction task, thus indicating that the respective mood inductions had the expected overall effects (i.e., the negative induction induced negative emotions and the positive induction induced positive emotions). Specifically, as expected, in the negative mood induction negative affect significantly increased post-induction and then gradually returned to baseline levels while positive affect significantly decreased. The opposite pattern was found with regards to the positive mood induction. Table 5 presents the results from pairwise comparisons for both negative and positive mood inductions using the Bonferroni correction.

**Table 5:** Results of pairwise comparisons of time based on estimated marginal means for 2(Group: NSSI vs. non-NSSI) X 4(Time: pre, post, 1 min post, 2 min post) repeated measures ANOVAs for negative and positive affect following a negative and positive mood induction.

	***M***	***SD***
**Negative affect—negative mood induction**		
Pre	14.35^a^	0.63
Post	17.40^b^	0.70
1 min post	15.88^c^	0.66
2 min post	14.12^a^	0.59
**Positive affect—negative mood induction**		
Pre	24.57^a^	0.85
Post	18.94^b^	0.68
1 min post	18.43^b, c^	0.72
2 min post	18.85^b, c, d^	0.77
**Negative affect—positive mood induction**		
Pre	13.71^a^	0.46
Post	12.10^b^	0.31
1 min post	12.09^b, c^	0.34
2 min post	12.47^b, c, d^	0.38
**Positive affect—positive mood induction**		
Pre	23.85^a^	0.82
Post	27.29^b^	1.07
1 min post	23.79^a^	0.98
2 min post	21.41^c^	0.92

*Significant differences in reports of affect over time points, as found using pairwise comparisons of estimated marginal means with the Bonferroni correction, are indicated by superscript letters within the column for means. Time points with the same superscript letter are not significantly different from one another*.

Meanwhile, in terms of main effects for Group (NSSI vs. non-NSSI), women with a history of NSSI did not report significantly different positive or negative affect compared to their non-NSSI peers in either mood induction task. Thus, although the respective mood inductions functioned as expected in terms of eliciting positive and negative affect, participants followed a similar pattern of response within each mood induction regardless of NSSI engagement.

## Discussion

The purpose of the present study was to investigate differences between individuals with and without a history of NSSI engagement on the experience of positive and negative emotions in terms of: (1) self-reported emotion reactivity, (2) self-reported emotion regulation, and (3) in-person experience of emotions in response to both a positive and negative mood induction. In what follows, the study's findings, limitations, and implications will be discussed.

Consistent with previous studies, the present results revealed that participants with a history of NSSI reported significantly greater difficulties in negative emotion reactivity than the comparison group on the self-report questionnaires (e.g., Gratz, 2006; Najmi et al., 2007; Jenkins and Schmitz, 2012). A similar pattern was found with respect to individuals' ability to regulate their negative emotions. Specifically, those with a history of NSSI reported significantly greater difficulties in regulating their negative emotions than those without such a history. These findings are consistent with previous studies examining the emotion reactivity and regulation of negative emotions in individuals with and without a history of NSSI (e.g., Gratz and Roemer, 2008; Heath et al., 2008; Peh et al., 2017; You et al., 2018).

However, contrary to hypotheses, no differences were found for self-reported reactivity or regulation of positive emotions between participants with and without a history of NSSI. Previous studies have examined the experiencing of positive emotions among individuals who engage in NSSI, with findings suggesting those who engage in NSSI report experiencing less positive emotion; however, results have been mixed (e.g., Bresin and Gordon, 2013; Arbuthnott et al., 2014; Santangelo et al., 2017). The present findings build on previous literature by going beyond the frequency of experiencing positive emotions, which is only one aspect of emotion reactivity, to establish a more comprehensive understanding of reactivity as it relates to NSSI. Specifically, in the present study, the *Emotion Reactivity Scale* (Nock et al., 2008) was adapted to assess positive emotions for a more complex assessment of emotion reactivity through individuals' sensitivity to emotions, their perception of emotion intensity, and the rate at which they experience persistence of emotions. Surprisingly, when using this more complex assessment, no differences in reactivity to positive emotions were found between women with and without engagement in NSSI on their perception of their emotion reactivity to positive emotions.

The discrepancy between self-reported positive and negative emotion reactivity and regulation is particularly interesting given that these assessments were conducted during the same session. This discrepancy suggests that women who engage in NSSI may perceive a difference in their tendency to react to or regulate positive vs. negative emotions. Specifically, although there are significant differences in the self-reported response to negative emotions between women with and without a history of NSSI engagement, there seem to not be significant differences when it comes to responding to positive emotions. These findings highlight the need to actively compare both positive and negative emotions when conducting research on NSSI. Additionally, further research is needed to assess self-reported positive emotion reactivity in both complex and simple ways in order to deepen our understanding of why individuals who self-injure report experiencing less frequent positive emotion when only assessing frequency of positive emotions (e.g., Victor and Klonsky, 2014) but report a comparable positive emotion reactivity when using a more complex assessment.

Uniquely, the current study simultaneously measured emotion reactivity and regulation through self-report and *in vivo* mood inductions of emotions with both valences. Contrary to what was hypothesized, results from the mood induction task indicated that, although the mood inductions functioned as expected for both women with and without a history of NSSI, no group differences emerged in reactivity or regulation for either negative or positive emotions as a function of NSSI engagement. Although theoretically this lack of significant differences may have been due to low power, this is unlikely given how similar the means are between the NSSI and no-NSSI groups for both types of affect in both mood inductions. Furthermore, this lack of group differences in response to the mood inductions was particularly surprising given the consistent findings of group differences in self-reports of negative emotion reactivity and regulation between individuals with and without a history of NSSI (e.g., Jenkins and Schmitz, 2012).

A potential explanation for this finding may be that self-reported differences in negative emotion reactivity and regulation are not reflective of actual differences in the regulatory processes of women with a history of NSSI. This would suggest that self-report assessments may be biased representations of what some women who engage in NSSI are actually experiencing emotionally. For example, some women who engage in NSSI may be particularly sensitive to the experience of a typical negative emotional response to stimuli and their sensitivity to that experience may cause them to feel that it is extremely intense when in fact it is comparable to their non-NSSI peers' experience. Therefore, their subjective interpretation of their negative emotional experience may be what is driving the self-reported differences in negative emotion reactivity and regulation.

Alternatively, the type of mood inductions selected may have had an impact on participants' response. Indeed, Arbuthnott and colleagues (Arbuthnott et al., 2014) found results conflicting with the current study's but used a rumination induction in which participants were asked to think about a personal experience that was upsetting to them and to describe why they felt the way they did about that situation. However, there may be a lack of standardization in this type of experimental task since it is possible that the personal experiences recalled by those with a history of NSSI in the induction were actually far more negative than those recalled by the non-NSSI comparison group. Meanwhile, the present results are consistent with Davis and colleagues (Davis et al., 2014) findings and both studies used mood inductions that were not related to participants' personal experiences (e.g., video clips were used). Thus, more research is needed to determine the potential impact of the type of mood induction used.

Interestingly, a recent mood induction study by Boyes and colleagues (Boyes et al., 2020) found that individuals with a history of NSSI did not report differences in self-reported negative emotion reactivity but reported significantly lower self-reported positive emotion reactivity when compared to those with no history of NSSI. Furthermore, when looking at positive and negative emotion reactivity in response to both a positive and a negative mood induction, individuals with a history of NSSI displayed lower emotion reactivity for both negative and positive emotions than those without a history of NSSI engagement.

However, a number of factors may account for the discrepancy between Boyes and colleagues' (Boyes et al., 2020) study and the current study. Most importantly, the measure of emotion reactivity for positive and negative emotions used by Boyes and colleagues (Boyes et al., 2020), the *Emotion Reactivity Intensity and Perseverance Scale* (Ripper et al., 2018), relied heavily on social comparison (i.e., “When exposed to a situation that would make the ‘average' person experience this feeling, how likely is it that you will experience this particular feeling?”) whereas the Emotion Reactivity Scale (Nock et al., 2008) used in the current study focused solely on participants' own experiences of positive and negative emotion reactivity. Therefore, future studies may need to simultaneously assess both self-focused experience of positive and negative emotions and other-focused experience based on social comparison.

Furthermore, differences in sample demographics may also account for this discrepancy in findings. Specifically, although the present study had an exclusively female sample of participants, Boyes and colleagues (Boyes et al., 2020) only had a majority of female participants (73.8%), which may have influenced findings given that studies have demonstrated potential gender differences in the experience of NSSI (e.g., Sornberger et al., 2012).

Finally, Boyes and colleagues (Boyes et al., 2020) chose to recruit participants with a lifetime history of NSSI while controlling for their history of mental illness; meanwhile, the present study focused on women who had engaged in NSSI over the past 2 years. This is particularly important because research shows that emotion reactivity and regulation may differ as a function of the recency of NSSI engagement (i.e., lifetime vs. current); therefore, this may have contributed to the differences in results obtained across both studies.

Despite differences in findings, these two recent mood induction studies strongly highlight the need for further research in the field of NSSI to better understand the differences between self-reported and *in vivo* emotion reactivity and regulatory processes while also clearly differentiating between positive and negative emotions. Overall, the results of the current study suggest that women who engage in NSSI may (a) interpret their self-reported emotion reactivity and regulation to be worse for negative emotions and comparable for positive emotions when compared to their non-NSSI peers; and (b) experience negative and positive emotions comparably to their non-NSSI peers in response to both a negative and positive mood induction. This suggests that women who engage in NSSI may be less reactive to negative situations and may be better at regulating their emotions (negative and positive) than they believe.

### Limitations and Future Directions

The current study is limited in the generalization of its findings to the investigation of emotion reactivity and regulation in female university students due to the insufficient number of men who engage in NSSI who volunteered to participate in this study. However, this is unfortunately a common limitation in NSSI research [e.g., see review by Cipriano et al. (2017)]. Future research is needed to investigate gender differences in NSSI research, which is particularly important given that findings show marked gender differences in preferred method of NSSI engagement with women engaging more in self-cutting and men engaging in self-hitting or burning (Sornberger et al., 2012). Similarly, the sample in the present study consisted of undergraduate students; therefore, further research on emotion reactivity and regulation for positive and negative emotions is needed to extend beyond using university samples.

Although the PANAS was used in the present study as a highly validated measure of emotional experiences, future studies should incorporate validity scales as well as a broader variety of assessments of state emotional experiences including visual analog scales and objective measures of mood reactivity. Similarly, although the videos for the current mood inductions were found to be effective and standardizable, they may not be generalizable to the same degree within clinical samples. Most importantly, future studies need to replicate the present study using autobiographical mood induction techniques given that, as noted in the discussion, a study by Arbuthnott and colleagues (Arbuthnott et al., 2014) using autobiographical mood induction techniques found significant differences based on NSSI engagement.

Finally, although the positive and negative mood inductions used in the current study functioned in the expected manner, with the positive induction eliciting positive emotions and vice versa, there was an unexpected yet interesting lack of significant group differences in response to the mood inductions between individuals with and without a history of NSSI engagement. In light of these findings, it may be of interest for future studies to replicate this design with a larger sample size to account for low power. Furthermore, future research should ask participants at the end of the mood induction task whether they felt that they reacted more or less strongly compared to others. This would allow for the assessment of participants' subjective experience of their emotional states in the same moment and on the same task as opposed to having a more generalized self-report assessment that was completed prior to the mood induction tasks. Additionally, a potential confound to be considered is alexithymia, which has been associated with NSSI engagement and may have led the NSSI group to have experienced different physiological reactions without being able to label them as such (e.g., Greene et al., 2020).

## Conclusion

Despite some limitations, the current study presents novel findings with important implications for future research and clinical practice in the area of NSSI. In particular, this study is an important first step in investigating the differences in emotion reactivity and regulation for both negative and positive emotions using self-report measures as well as *in vivo* mood induction. The findings of the current study suggest that, despite self-reported differences, individuals with a history of NSSI may not differ from individuals who have not engaged in NSSI when experiencing negative and positive stimuli. Consequently, these results suggest implications about the need to consider that the focus in a clinical context should be less on changing emotion regulatory processes and more on accepting or tolerating emotional responses. Future research is needed to replicate these findings and extend our understanding of positive and negative emotion reactivity and regulation for individuals who engage in NSSI.

## Data Availability Statement

The raw data supporting the conclusions of this article will be made available by the authors, without undue reservation.

## Ethics Statement

The studies involving human participants were reviewed and approved by McGill University Research Ethics Board Office. The patients/participants provided their written informed consent to participate in this study.

## Author Contributions

MS and NH designed the study and organized the data collection. JM, MS, and NH conducted literature searches and formulated the research questions for the study. JM conducted the statistical analyses. MS, JM, SL, and NH contributed to the first full draft of the manuscript. JM and NH completed subsequent numerous edits and revisions. All authors contributed to the article and approved the submitted version.

## Conflict of Interest

The authors declare that the research was conducted in the absence of any commercial or financial relationships that could be construed as a potential conflict of interest.
